# Inequalities in catastrophic health expenditures in conflict-affected areas and the Colombian peace agreement: an oaxaca-blinder change decomposition analysis

**DOI:** 10.1186/s12939-021-01555-7

**Published:** 2021-09-29

**Authors:** Sebastián León-Giraldo, Juan Sebastián Cuervo-Sánchez, Germán Casas, Catalina González-Uribe, Noemi Kreif, Oscar Bernal, Rodrigo Moreno-Serra

**Affiliations:** 1 Alberto Lleras Camargo School of Government, Universidad de Los Andes, Carrera 1 No 19 – 27, Bloque Aulas, tercer piso, Bogotá, Colombia; 2 Interdisciplinary Centre of Development Studies, Universidad de Los Andes, Bogotá, Colombia; 3 School of Medicine, Universidad de Los Andes, Bogotá, Colombia; 4 Fundación Santa Fe de Bogotá University Hospital, Bogotá, Colombia; 5 Centre for Health Economics, University of York, York, UK

**Keywords:** Colombia, Peace treaty, Catastrophic expenditures, Health inequalities

## Abstract

**Background:**

The present study analyzes inequalities in catastrophic health expenditures in conflict-affected regions of Meta, Colombia and socioeconomic factors contributing to the existence and changes in catastrophic expenditures before and after the sign of Colombian Peace Agreement with FARC-EP guerilla group in 2016.

**Methods:**

The study uses the results of the survey *Conflicto, Paz y Salud *(CONPAS) conducted in 1309 households of Meta, Colombia, a territory historically impacted by armed conflict, for the years 2014 and 2018. We define catastrophic expenditures as health expenditures above 20% of the capacity to pay of a household. We disaggregate the changes in inequalities in catastrophic expenditures through the Oaxaca-Blinder change decomposition method.

**Results:**

The incidence of catastrophic expenditures slightly increased between 2014 to 2018, from 29.3 to 30.7%. Inequalities in catastrophic expenditures, measured through concentration indexes (CI), also increased from 2014 (CI: -0.152) to 2018 (CI: -0.232). Results show that differences in catastrophic expenditures between socioeconomic groups are mostly attributed to an increased influence of specific sociodemographic variables such as living in rural zones, being a middle-aged person, living in conflict-affected territories, or presenting any type of mental and physical disability.

**Conclusions:**

Conflict-deescalation and the peace agreement may have facilitated lower-income groups to have access to health services, especially in territories highly impacted by conflict. This, consequently, may have led to higher levels of out-of-pocket expenditures and, therefore, to higher chances of experiencing catastrophic expenditures for lower-income groups in comparison to higher-income groups. Therefore, results indicate the importance of designing policies that guarantee access to health services for people in conflict -affected regions but also, that minimize health care inequalities in out-of-pocket payments that may arouse between people at different socioeconomic groups.

## Background

For almost 60 years, Colombia has experienced one of the most long-lasting armed conflicts in the world. Colombia’s conflict has resulted in approximately 262,000 deaths, 80,000 forced disappearances, 15,000 victims of sexual assault, and more than 7 million internally displaced people [1, 2]. Colombia's conflict has affected in different ways and at various levels of intensity, several regions of Colombia, specific communities, and some of the most vulnerable population groups [3]. Nevertheless, these armed struggle's consequences go beyond the direct impacts on security and losses in human lives and influence other dimensions of social, political, and economic outcomes [4].

One of the dimensions where conflict has had important consequences is in health. Conflict, directly and indirectly, impacts health outcomes and opportunities [5]. Asides from general physical and mental health consequences, conflicts worsen health provision, and complicate health services operations and processes [6]. These effects are a consequence of direct damages to health facilities and health care infrastructures, conflict-related threats to health professionals, or structural problems such as inadequate institutions for promoting people’s rights and opportunities [7]. Health services may be forced to stop or to operate under challenging circumstances, which, in the long run, may lead health services to be unstable or rugged to maintain operations overtime [8].

In some regions, inadequate health services or difficulties to access these health care services translate into high costs for the individuals and difficulties in the coverage and the financial sustainability of health care systems [9]. Health systems worldwide usually appeal to public resources, taxes, or pre-payment services to finance health care [10]. Nevertheless, some of these health expenses are charged directly to healthcare users both as a mechanism for facilitating health financing and limiting moral hazard problems in health [11]. These direct payments covered by individuals and not the health system, such as certain medicines or specific health services, are usually called out-of-pocket expenses (OOPE).

OOPE may be an adequate mechanism for transferring some of the costs of health provision to the people that benefit from these services. Nevertheless, when these expenses surpass people’s capacity to pay, these costs become catastrophic expenses or “payments that exceed a household’s ability to pay, once food and basic consumption costs are deducted” [12]. In the long run, high OOPEs and continuous catastrophic expenditures may lead to financial ruin or difficulties in maintaining an adequate quality of life [13].

The presence of catastrophic expenditures in a region or a country usually reflects several types of economic system difficulties that restrain or limit economic development [14]. No matter the level of out-of-pocket expenditures, households are at risk of incurring in catastrophic payments if there are high levels of poverty, some social groups are excluded from financial risk protection mechanisms, and health care utilization is high, situations that are common in middle and low-income countries [15].

People experiencing financial difficulties or suffering from poverty are significantly affected by these circumstances. They may be more vulnerable to adverse health shocks and, ultimately, greater health care costs [16]. Even though there is a growing use of health care systems, developing countries usually have weak social institutions, inadequate risk pooling mechanisms, and inadequate tax-financed health care systems, limitations that manifest themselves in high levels of household health care expenditures [15]. These problems may be more severe in conflict-affected territories where households have financial limitations to generate income, and simultaneously, health provision is problematic within largely unregulated health markets [17]. The risk of experiencing bad health in conflict-affected regions increases as well as the financial burdens and limitations to which people may be exposed to, such as loss of jobs, destruction of public infrastructure in their communities (including health facilities) and, ultimately, greater risks of being sick [18]. If, simultaneously, these people should cover expensive health care treatments or belong to highly vulnerable population groups, who may have specific limitations to generate stable income streams, like elderly people or with adverse health conditions, catastrophic expenditures may lead to long-run financial ruin [19].

The health and financial risks outlined above may be distributed heterogeneously across the population and may raise equity concerns. Exposure to different levels of conflict incidence across different socioeconomic groups may lead to differences in the incidence of health payments and, ultimately, in the risk of experiencing catastrophic health expenditures. Moreover, in conflict-affected regions there is often variations in the exposure to conflict violence, leading to inequalities in health expenditures. Ultimately, health expenditures may impose greater financial burdens over certain socioeconomic groups, leading to inequities in health, and imbalances in overall quality of life and wellbeing [20]. Analyzing inequalities in catastrophic expenditures is essential to identify contributing factors that sustain these differences over time and, therefore, important to improve the design of public policies that reduce health financing disparities.

In 2016, Colombia signed a peace accord with *Fuerzas Armadas Revolucionarias de Colombia* (FARC-EP), one of the guerilla groups that, for years, dominated several of the territories of the Meta region [21]. The treaty led to the establishment of the *Espacios Territoriales de Capacitación y Reincorporación* (ETCR), created to facilitate the gradual reincorporation of demobilized guerilla groups to civil society. These processes, initially, may have contributed to a reduction in direct conflict violence, which may have led to a reduction of the physical and psychological consequences that direct armed struggle has on health outcomes, and simultaneously, to reductions in health expenditures. Nevertheless, conflict’s health consequences may have long-run impacts and may be more severe in certain population groups that are more impacted by armed conflict.

Colombian health care system is characterized by two simultaneous health regimes that cover most of the population. The subsidized regime covers health care costs of people that are unemployed or that face specific barriers to access the system, based in a pre-defined portafolio of minimum health care services. The contributive regime is constituted by people that are currently employed, where specific ratios of health care costs are covered by the employee and others by the contractors. Health services are delivered by several health services providers, and health resources are administered by health entities (*Entidades Promotoras de Salud – EPS* in Spanish) which are obligated to cover the health needs of the population that is affiliated to the institution.

To contribute to our understanding of the issues above, our study investigates the evolution of catastrophic expenditures over time and between socioeconomic groups in the Colombian region of Meta, an area that was intensely affected by conflict violence, mostly related to actions of FARC-EP. To evaluate the prevalence and change of inequalities in health expenditures, we measure the change in the incidence of catastrophic expenditures for the years 2014 and 2018 and analyze, through a decomposition method, the extent to which differences in specific socioeconomic factors contribute to inequalities in health expenditures in each period. First, we present our methodological approach, followed by our main results and concluding with a discussion of our findings.

## Methods

Our research uses primary data from the survey *Conflicto, Paz y Salud* (CONPAS), designed by our research team. The survey was conducted in year 2018 in 1309 households of the department (province) of Meta, Colombia and includes responses for year 2014 collected through recall questions answered by survey respondents. The survey was conducted in the department of Meta due to certain social and historical characteristics of these territory. First, the region has been, historically, one of the departaments most affected by conflict violence in Colombia. However, at the same time, conflict intensity has highly varied between different municipalities of Meta, with some experiencing low or minimum levels of conflict violence and others experiencing high levels of conflict violence. Consequently, this region is an appropriate location to measure the relationship between different levels of conflict incidence and the chances of households located in these territories to experience catastrophic expenditures.

The survey sample was selected through a probabilistic method using a two-stage sampling approach, being representative at the level of rural and urban areas and for groups of Meta municipalities affected by different levels of conflict violence (see below). Urban areas are defined in Colombia as a group of buildings and contiguous structures, grouped in blocks, delimited in streets and avenues; while, rural areas are characterized by the dispersed disposition of housings and/or agricultural territories [22]. A survey company was hired to perform data collection and surveyers were trained to pose recall questions for year 2014. Through the utilization of specific worldwide events which occurred in 2014, highly recognized in the Colombian context (specifically, the Soccer World Cup of Brazil of 2014), surveyers were trained to help participants recall specific periods of time and information that developed during the year 2014 of Brazil's Soccer World Cup. All households (N:1309) responded to the survey and answered all question posed by surveyers.

To calculate the incidence of catastrophic expenditures, we use the methodology proposed by Xu [23]. A household incurs catastrophic expenditures if the ratio of out-of-pocket expenditures over a household's capacity to pay exceeds a certain threshold. In the literature, this threshold ranges between 20-40% and is usually established depending on a country's income level. We use a threshold of 20% to make our results comparable with previous national studies conducted in Colombia about catastrophic expenditures [24, 25]. Out-of-pocket expenditures were directly measured by asking respondents what amount of the total household expenditure in the previous month was used to pay for health-related services or products, including health insurance copayments but excluding insurance premia. Capacity to pay is defined as the household's available expenditure capabilities once minimum subsistence expenditures are deducted – the difference between total expenditure and minimum required food expenditures [23]. Total household expenditure was measured directly in the survey. Minimum required food expenditures are calculated following the procedure proposed by Xu [23].

We first conduct some descriptive analysis of our data. Then, we use a multivariate logistic regression to analyze sociodemographic factors that affect the risk to experience catastrophic health expenditures for both 2014 and 2018. Sociodemographic factors included in logistic regressions refer to the characteristics of the head of the household, individual who responded the survey in each of the 1309 households of the study.

Certain variables were grouped in categories to facilitate analysis. Work was grouped in people with formal jobs (with any type of working contract either from a company or the Government), people out of the labor force (students, retired people, and people with disabilities), and informal jobs (other types of jobs with no formal contracting). Health insurance schemes represent current affiliation models in Colombian Health System: subsidized, people who receive health services subsidied by public officials, contributive, people who are covered by their employees, other special schemes currently in existence (health coverage tailored to militaries or certain population groups) and people not affiliated in the system. Education levels are grouped in primary school (1^st^ to 5^th^ grade finished education), secondary level (6^th^ to 11^th^ grade finished education) and undergraduate (first level technical or university education; no respondents have postgraduate studies). Finally, ethnicity is categorized in people from mayor ethnic groups (white or mestizo) and minor (other ethnic groups).

We estimate inequalities in catastrophic expenditures by calculating concentration curves and the health concentration index (HCI) for both years, 2014 and 2018. A concentration curve plots the cumulative percentage of a variable (in our study catastrophic expenditures) in the y-axis, against the cumulative percentage of the population ranked from poorest to richest, measured through a specific socioeconomic ranking variable, in the x-axis [26]. The concentration curve allows us to measure if catastrophic expenditures are disproportionally occurring in a specific socioeconomic group and, consequently, if inequalities in health outcomes are present in this population. The HCI, defined as twice the area between the concentration curve and the perfect equality line, measures how severe this inequality is. A HCI of zero represents perfect equality, below zero that inequality is against the poor, and above zero that inequality is against the rich. To calculate these HCIs, we estimate, with data of household characteristics, the Household Wealth Index (HWI) [27] which is a measure of household non-monetary income based on the possession of different assets.

We estimate the HCI for catastrophic health expenditures for both years 2014 and 2018. We then analyze to what extent changes in inequalities in catastrophic expenditures between 2014 and 2018 may be explained by changes in inequalities in sociodemographic variables related to health expenditures. Two mechanisms may explain changes in inequalities in catastrophic expenditures: a change in the distribution of an explanatory variable across socioeconomic groups between these two time periods (changes in distribution) or changes in the influence of a socioeconomic variable over time (changes in marginal effects). To separate these two components, we use the Oaxaca-Blinder decomposition method [28], which separates changes in the health concentration index between two time periods as a linear function of the changes in explanatory socioeconomic variables.

Socioeconomic factors for the decomposition analysis were initially selected through a literature review to identify variables correlated with catastrophic expenditures in previous studies [29, 30] and, more specifically in previous research in post-conflict settings [17] which allowed to select appropriate explanatory variables. In the final decomposition model, the following variables were included: gender, age, residence (urban or rural), currently experiencing internal displacement, type of work, ethnicity, marital status, education level, conflict incidence of the municipality of residence, number of household members, number of children under six years old, health insurance scheme, being recently sick or hospitalized, and measurements of possible mental health disorders and mental or psychical disabilities. Internal displacement status was self-reported by survey respondents. Possible mental health disorder was measured using the Self Report Questionnaire (SRQ-20) [31], and physical and mental health disabilities were measured through the World Health Organization Disability Assessment Schedule (WHODAS). The municipality's conflict incidence was estimated using the classification developed by the Conflict Analysis Resource Center [32], with municipalities categorized as being with “high” , “low” or “no conflict”, in addition to Meta’s largest capital city (Villavicencio), mostly highly affected, but analyzed separately because of its size in comparison to other municipalities.

All calculations were conducted using robust standard errors and 95% confidence intervals. We estimate the models using the software Stata 15 MP, using previously assigned survey sample weights.

## Results

Table 1 shows a summary of the characteristics of study participants and the incidence of catastrophic expenditures in the household using a threshold of 20%. Sociodemographic data displayed in Table 1 corresponds to the information of the head of the household, person who reported all information in the survey. The mean of the continuous variables is reported, specifically, the average number of children under five years old and the average number of people living per household (N:1309 sample in both years).

**Table 1:** Descriptive Statistics, CONPAS 2014 – 2018

Variable	CONPAS 2014 (N:1309)	CONPAS 2018 (N:1309)
	N (%)	N (%)
*Concentration index*	-0.151	-0.233
*Catastrophic expenditure*		
No	925 (70.7)	907 (69.3)
Yes	384 (29.3)	402 (30.7)
*Age*		
Under 18 years	63 (4.8)	0 (0.0)
18 – 44 years	639 (48.8)	598 (45.7)
45 – 64 years	468 (35.7)	506 (38.7)
65 years or more	139 (10.6)	205 (15.6)
*Gender*		
Male	600 (45.8)	600 (45.8)
Female	709 (54.2)	709 (54.2)
*Residence*		
Urban	782 (59.7)	782 (59.7)
Rural	527 (40.3)	527 (40.3)
*Internal displacement*		
No	759 (58.0)	759 (58.0)
Yes	550 (42.0)	550 (42.0)
*Work type*		
Formal	280 (21.4)	204 (15.6)
Informal	906 (69.2)	977 (74.6)
Out of Labor Force	123 (9.4)	128 (9.8)
*Marital status*		
Married	284 (21.7)	281 (21.5)
Consensual Union	564 (43.1)	536 (40.9)
Divorced	220 (16.8)	299 (22.8)
Widow/er	72 (5.5)	94 (7.2)
Single	169 (12.9)	99 (7.5)
*Conflict level*		
Lived outside Meta	163 (12.4)	0 (0.0)
Capital city	300 (22.9)	300 (22.9)
Highly affected	150 (11.4)	306 (23.4)
No conflict	188 (14.3)	294 (22.5)
Lowly affected	508 (38.8)	409 (31.2)
*Ethnicity*		
Majority	1027 (78.5)	1027 (78.5)
Minority	282 (21.5)	282 (21.5)
*Education*		
None	79 (6.0)	79 (6.0)
Primary School	564 (43.1)	535 (40.9)
Secondary School	454 (34.7)	439 (33.5)
Undergraduate	212 (16.2)	256 (19.6)
*SRQ+*		
No	1111 (84.9)	885 (67.6)
Yes	198 (15.1)	424 (32.4)
*Sick in the previous 12 months*		
No	728 (55.6)	525 (40.1)
Yes	581 (44.4)	784 (59.9)
*Health insurance scheme*		
Not affiliated	68 (5.2)	70 (5.3)
Contributive	410 (31.3)	350 (26.7)
Subsidized	771 (58.9)	829 (63.3)
Other (exception, special schemes)	60 (4.6)	60 (4.6)
*Children under 5 years old (mean)*	0.267	0.267
*Log household size (mean)*	1.078	1.078
*WHODAS (mean)*	3.128	4.735

Source: Own elaboration based on CONPAS 2014 – 2018

*Abbreviations*: *SRQ+* person has a possible mental health disorder (i.e., a SRQ positive case), *WHODAS* Disability score in World Health Organization Disability Assessment Schedule

In 2018 all people surveyed had the legal age (18 years or over) and lived in Meta, but, in 2014, year for which retrospective questions were conducted, some of these people were minors or lived in urban or rural areas outside of Meta. In the first row of the age variable, this small group (N:63) is also considered.

The greatest group of people surveyed is 18-44 years old both in 2014 (51.8%) and in 2018 (45.7%). Most people are women (54.2%) and live in urban areas (59.7%). No differences are reported in zone of residence (urban or rural) between 2014 and 2018, as this question was only answered in 2018. Approximately 42.0% of the population report being a victim of internal displacement. The majority of people work in informal jobs, both in 2014 (69.2%) and 2018 (74.6%), live in lowly conflict-affected territories (38.8% in 2014 and 31.2% in 2018), and on average, 78.5% belong to an ethnic group considered majority in the territory (mestizo and white). Most of the population surveyed attained up to primary school education (43.1 and 40.9%). Finally, 15.1 and 32.4% of people have a possible mental health disorder, measured through the Self Report Questionnaire, indicated, in this paper as SRQ+ (i.e a SRQ positive case). Approximately 29.3 and 30.7% of households faced catastrophic health expenditures in 2014 and 2018, respectively. The average number of children under five years is the same for both years, as the question was not included as a recall question for 2014, but was included in the model due to its correlation with catastrophic expenditures in previous studies [17]

### Logistic regression analysis

Table 2 shows the odds ratios (OR), calculated from a multivariate logistic regression, to analyze sociodemographic factors that increase the risk of specific populations to have catastrophic expenditures for 2014 and 2018. OR quantify the association between “exposure” to a specific factor and an outcome, representing the odds that this outcome occurs given a particular exposure, compared to the odds of the outcome occurring in the absence of that exposure [33].

**Table 2:** Logistic regression model for catastrophic expenditures, 2014 and 2018

Variable	OR (2014)	OR (2018)
*Internal displacement*		
No	1.00	1.00
Yes	1.33* [1.01-1.75]	1.20 [0.91-1.59]
*Age group*		
Less than 18 years	1.77 [0.87-3.61]	N. A
18 – 44 years	1.00	1.00
45 – 64 years	1.19 [0.86-1.65]	1.39* [1.01-1.92]
65 years or more	1.99** [1.18-3.34]	1.68* [1.05-2.70]
*Gender*		
Male	1.00	1.00
Female	0.99 [0.75-1.30]	1.21 [0.91-1.61]
*Residence*		
Urban	1.00	1.00
Rural	1.54** [1.13-2.10]	1.47* [1.03-2.09]
*Work*		
Formal	1.00	1.00
Informal	0.99 [0.67-1.46]	1.38 [0.88-2.18]
Out of Labor Force	0.61 [0.33-1.11]	1.34 [0.75-2.41]
*Conflict level*		
Lived outside Meta	1.38 [0.86-2.22]	
Capital city	1.00	1.00
Highly affected	1.50 [0.90-2.51]	2.80** [1.74-4.49]
No conflict	1.43 [0.91-2.26]	1.55 [0.99-2.42]
Low conflict intensity	1.50* [1.02-2.20]	2.20** [1.45-3.36]
*Ethnicity*		
Majority	1.00	1.00
Minority	0.90 [0.66-1.23]	1.03 [0.76-1.40]
*Education*		
None	1.00	1.00
Primary School	1.32 [0.76-2.32]	0.83 [0.49-1.42]
Secondary School	1.21 [0.65-2.26]	0.83 [0.46-1.51]
Undergraduate	1.56 [0.78-3.13]	1.20 [0.62-2.32]
*Marital status*		
Married	1.00	1.00
Consensual Union	0.98 [0.69-1.38]	0.83 [0.59-1.18]
Divorced	0.90 [0.58-1.38]	0.77 [0.51 - 1.16]
Widow/er	1.10 [0.60-2.03]	0.68 [0.38 -1.22]
Single	0.73 [0.42-1.27]	0.51* [0.26-0.97]
*Health insurance scheme*		
Not affiliated	1.09 [0.59-2.02]	1.24 [0.66-2.33]
Contributive	1.00	1.00
Subsidized	0.74 [0.51-1.05]	0.77 [0.52-1.12]
Other	1.52 [0.83-2.79]	1.83 [0.98-3.39]
*Sick in the previous 12 months*		
Yes	1.00	1.00
No	0.61** [0.47-0.80]	0.63** [0.47-0.83]
*Children under 5 years old*	1.09 [0.84-1.42]	1.02 [0.77-1.33]
*Household size*	1.11 [0.84-1.46]	1.04 [0.77-1.38]
*WHODAS*	1.06** [1.03-1.09]	1.04** [1.01-1.06]
*SRQ+*		
No	1.00	1.00
Yes	1.14 [0.78-1.67]	1.20 [0.88-1.65]
*Poverty quintiles*		
Quintile 1	1.00	1.00
Quintile 2	0.98 [0.66-1.47]	0.94 [0.62-1.41]
Quintile 3	0.75 [0.48-1.16]	0.91 [0.57-1.47]
Quintile 4	1.13 [0.70-1.83]	0.60 [0.35-1.04]
Quintile 5	0.78 [0.46-1.35]	0.82 [0.45-1.49]

Source: Own elaboration based on CONPAS 2014 – 2018 (** *p* value<=0.01; * *p* value <=0.05)

*Abbreviations*: *SRQ+*: person has a possible mental health disorder (i.e., a SRQ positive case), *WHODAS* Disability score in World Health Organization Dissability Assessment Schedule

For 2014, elderly people (65 years or more) had a higher chance (OR: 1.99) of experiencing catastrophic expenditures, in comparison to the younger population group. Similarly, people in rural zones have higher chances of experiencing catastrophic expenditures (OR:1.68), in comparison to people living in urban areas. People with any type of physical or mental disability have 1.06 higher chances of experiencing catastrophic expenditures than others without these health conditions. People living in municipalities with low conflict intensity had a higher chance (OR: 1.50) of incurring catastrophic expenditures than people living in the capital city. Internally displaced people had a higher chance (OR: 1.33) of experiencing catastrophic expenditures than those not displaced. Finally, people who did not had any type of illness in the previous 12 months had a fewer chance (OR: 0.61) of facing catastrophic expenditures.

In 2018 similar socioeconomic factors contributed to a higher chance of experiencing catastrophic health expenditures, albeit with slight differences. Older people (OR:1.68) and middle-aged people in the 45-64 age range (OR: 1.38) had higher chance of experiencing catastrophic health expenditures than the younger age group. People in both low and highly affected areas had a higher chance (OR: 2.20 and 2.80, respectively) of experiencing catastrophic health expenditures than people living in other municipalities. Finally, people with any type of physical or mental disability (measured with WHODAS) had a higher chance of experiencing catastrophic expenditures (OR: 1.04). On the contrary, those who did not experience any type of health problem in the previous 12 months had a lower chance of experiencing catastrophic expenditures (OR: 0.63).

### Oaxaca decomposition analysis

**Fig. 1:**
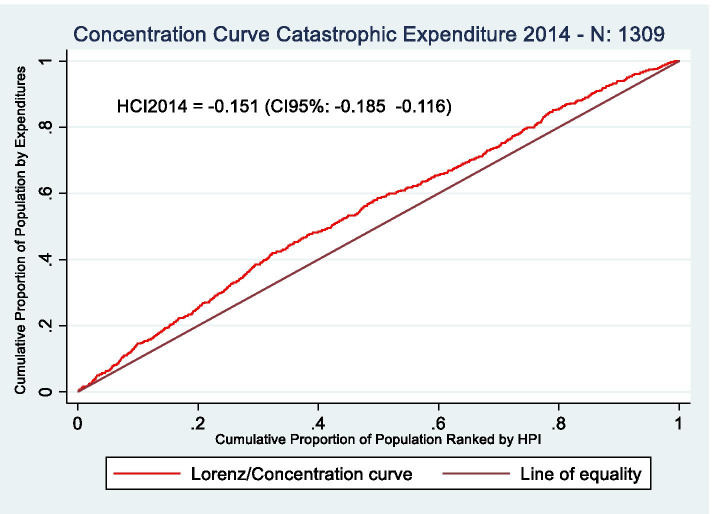
Concentration curves for catastrophic expenditures, 2014. Source: Own elaboration based on CONPAS 2014. Abbreviations: HCI: Health concentration index; CI: confidence interval; HPI: Household poverty index

**Fig. 2:**
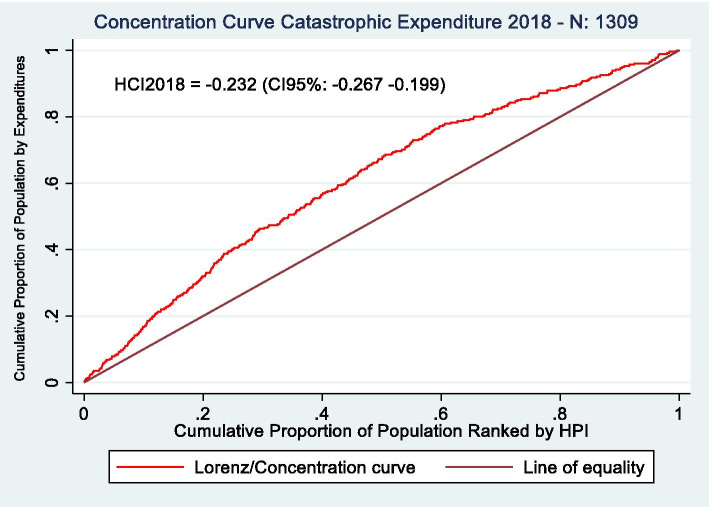
Concentration curves for catastrophic expenditures, 2018. Source: Own elaboration based on CONPAS 2018. Abbreviations: HCI: Health concentration index; CI: confidence interval; HPI: Household poverty index

Even though the incidence of catastrophic health expenditures is similar between 2014 and 2018 (29.3 and 30.7%), there are differences in the incidence of these expenditures between socioeconomic groups. Figures 1 and 2 show concentration curves for catastrophic expenditures for both 2014 and 2018.

Inequalities in catastrophic health expenditures were higher in 2018 than in 2014. For 2014 and 2018, the health concentration indexes (HCI) are, respectively, -0.152 and -0.232 (Figs. 1 and 2), which means that catastrophic health expenditures were unevenly distributed among different socioeconomic levels and, more specifically, that inequalities against the poorest groups were observed in both years.

The change in the slope of the concentration curve shows the areas where inequalities in catastrophic expenditures are growing faster. For the year 2018, more people in the 0-20% income group are experiencing catastrophic expenditures compared to 2014, as shown by the higher slope of the concentration curve in the 0-20% of the x-axis. Catastrophic expenditures in 2018 reached its peak at the 30% percentile, point at which the cumulative percentage of catastrophic expenditures is of 50% and, for 2014 is, approximately 42%. In the 30-50% income group, the rate of people with catastrophic expenditures grows at similar rates for both years. Nevertheless, while in the 50-70% income group, there is a steady reduction in the number of people with catastrophic expenditures for the year 2018, in 2014, catastrophic expenditures continue growing at similar rates to those found at the lower-income groups. In the 80-100% income group, catastrophic expenditures grow at much lower rates for 2014 and 2018 than the most impoverished populations.

In summary, higher inequalities in 2018 are mostly explained by higher levels of catastrophic expenditures at the lower and middle-income groups (Quintiles 1 through 3), in comparison to 2014. Inequalities in 2018 increase even more due to a reduction in catastrophic expenditures in the 50-70% (Quintile 4), in comparison to 2014.

Table 3 shows the results of applying the Oaxaca decomposition method to identify the socioeconomic factors contributing to changes in inequalities in catastrophic expenditures. The Oaxaca method decomposes the change in the health concentration index into changes in the distribution of determinant factors (distributional effect in Table 3) and changes in the influence of a socioeconomic variables over time (coefficient effect in Table 3). In our table, the term ηΔC represents changes in the levels or values of a socioeconomic variable between socioeconomic groups from 2014 to 2018 (distributional effect), while the term CΔη captures changes in the influence of a specific variable for determining health inequalities between these two years (coefficient effect).

**Table 3:** Oaxaca-blinder decomposition of changes in inequalities of catastrophic expenditures, 2014-2018

Variable	Distributional effect	Coefficient effect	Total
	ηΔC	CΔη	
*Displaced*	-	-	-
No	-	-	-
Yes	0.002	0.010	0.012
*Age group*	-	-	-
Less than 18 years	NA	-0.001	-0.001
18 – 44 years	-	-	-
45 – 64 years	0.001	-0.006	-0.005
65 years or more	-0.002	-0.000	-0.002
*Gender*	-	-	-
Male	-	-	-
Female	0.003	0.006	0.009
*Residence*	-	-	-
Urban	-	-	-
Rural	-0.013	0.010	-0.003
*Work*	-	-	-
Formal	-	-	-
Informal	0.013	-0.067	-0.054
Out of Labor Force	-0.002	0.017	0.015
*Conflict level*	-	-	-
Outside Meta	0.000	-0.002	-0.002
Capital city	-	-	-
Highly affected	-0.007	-0.050	-0.057
No conflict	0.002	0.004	0.006
Lowly affected	0.014	-0.012	0.002
*Ethnicity*	-	-	-
Majority	-	-	-
Minority	0.000	-0.005	-0.005
*Education*	-	-	-
None	-	-	-
Primary School	-0.002	0.056	0.054
Secondary School	0.004	-0.020	-0.016
Undergraduate	-0.000	-0.013	-0.013
*Marital status*	-	-	-
Married	-	-	-
Consensual Union	-0.005	0.003	-0.002
Divorced	0.003	0.004	0.007
Widow/er	-0.002	0.003	0.001
Single	0.000	-0.001	-0.001
*Health insurance scheme*	-	-	-
Not affiliated	0.000	0.000	
Contributive	-	-	-
Subsidized	-0.006	-0.009	-0.015
Other	0.001	0.002	0.003
*Sick in the previous 12 months*	-	-	-
Yes	-	-	-
No	-0.004	0.008	0.004
*Children under 5 years old*	0.000	-0.001	-0.001
*Log household size*	0.001	-0.001	0.000
*WHODAS*	0.003	0.000	0.003
*SRQ+*	-	-	-
No	-	-	-
Yes	0.003	-0.005	-0.002
*Quintiles*	-	-	-
Quintil 1	-	-	-
Quintil 2	0.000	0.003	0.003
Quintil 3	-0.000	0.000	-0.000
Quintil 4	-0.000	-0.038	-0.038
Quintil 5	0.000	0.004	0.004
*Total*	**-0.007**	**-0.104**	**-0.111**

*Source*: Own elaboration based on CONPAS 2014 – 2018

*Abbreviations SRQ+* person has a possible mental health disorder (i.e., a SRQ positive case), *WHODAS* Disability score in World Health Organization Dissability Assessment Schedule

The decomposition results for changes in catastrophic expenditure inequalities show that most of the differences in inequalities over time are explained as a consequence of changes in the influence, i.e. of sociodemographic characteristics for explaining health expenditures inequalities (-0.104), instead of changes in the distribution of levels of these determinants (-0.007). Working in the informal sector (-0.067) and living in municipalities highly affected by conflict violence (-0.050) are the two factors with the highest individual contributions to the increase in catastrophic health expenditure inequalities, related to changes in the influence over time. Changes in the distribution of populations between rural and urban residence areas (-0.018) are the major contributors to changes in catastrophic expenditure inequalities because of a distribution effect.

### Robustness check

In Table 4, we perform a robustness check of the decomposition results using different threshold levels for defining catastrophic expenditures. Results show that while the distributional effects are mostly robust to the threshold used, the coefficient effect estimates vary more widely with the specific threshold adopted. These changes could mostly be attributed to other variables that start to be relevant to explain the incidence of catastrophic expenditures at specific levels, changing the weight of certain coefficients at specific thresholds. Nevertheles, results show that the relative importance of most variables continues to be consistent along different thresholds.

**Table 4:** Robustness check – Oaxaca decomposition at different thresholds

	5%	10%	15%	25%	30%
**Variable**	**Distributional effect – ηΔC**				
*Displaced*	**-**	**-**	**-**	**-**	**-**
No	**-**	**-**	**-**	**-**	**-**
Yes	0.002	0.002	0.002	0.000	-0.001
*Age group*	**-**	**-**	**-**	**-**	**-**
Less than 18 years	0.000	0.000	0.000	0.000	0.000
18 – 44 years	-	-	-	-	-
45 – 64 years	0.000	0.000	0.001	0.001	0.001
65 years or more	-0.001	-0.001	-0.002	-0.002	-0.003
*Gender*	**-**	**-**	**-**	**-**	**-**
Male	**-**	**-**	**-**	**-**	**-**
Female	0.001	0.002	0.002	0.000	0.001
*Residence*	**-**	**-**	**-**	**-**	**-**
Urban	**-**	**-**	**-**	**-**	**-**
Rural	-0.010	-0.013	-0.014	-0.013	-0.016
*Work*	**-**	**-**	**-**	**-**	**-**
Formal	**-**	**-**	**-**	**-**	**-**
Informal	0.012	0.006	0.008	0.004	-0.001
Out of Labor Force	-0.002	-0.002	-0.002	-0.002	-0.000
*Conflict level*	**-**	**-**	**-**	**-**	**-**
Outside Meta	0.000	0.000	0.000	0.000	0.000
Capital city	**-**	**-**	**-**	**-**	**-**
Highly affected	-0.006	-0.006	-0.007	-0.008	-0.009
No conflict	0.003	0.002	0.002	0.001	0.001
Lowly affected	0.013	0.013	0.012	0.015	0.014
*Ethnicity*	**-**	**-**	**-**	**-**	**-**
Majority	**-**	**-**	**-**	**-**	**-**
Minority	-0.000	-0.000	-0.000	-0.000	-0.000
*Education*	**-**	**-**	**-**	**-**	**-**
None	**-**	**-**	**-**	**-**	**-**
Primary School	0.001	0.001	**-**0.002	-0.001	-0.002
Secondary School	0.000	0.000	0.005	0.003	0.002
Undergraduate	-0.001	-0.001	-0.000	-0.000	-0.000
*Marital Status*	**-**	**-**	**-**	**-**	**-**
Married	**-**	**-**	**-**	**-**	**-**
Consensual Union	-0.002	0.000	-0.000	-0.009	-0.011
Divorced	0.002	0.001	0.002	0.004	0.005
Widow/er	-0.000	0.000	-0.001	-0.001	-0.001
Single	-0.000	-0.000	-0.000	-0.000	-0.000
*Health insurance scheme*	**-**	**-**	**-**	**-**	**-**
Not affiliated	-0.000	-0.000	-0.000	-0.000	-0.000
Contributive	**-**	**-**	**-**	**-**	**-**
Subsidized	-0.008	-0.006	-0.006	-0.004	-0.000
Other	0.000	0.001	0.000	0.002	0.002
*Sick in the previous 12 months*	**-**	**-**	**-**	**-**	**-**
Yes	-	**-**	**-**	**-**	**-**
No	-0.004	-0.004	-0.004	-0.004	-0.005
*Children under 5 years old*	0.001	0.001	0.000	0.000	0.000
*Log household size*	0.000	0.000	0.001	0.005	0.005
*WHODAS*	0.002	0.003	0.003	0.003	0.003
*SRQ+*	**-**	**-**	**-**	**-**	
No	**-**	**-**	**-**	**-**	**--**
Yes	0.001	0.001	0.002	0.003	0.005
*Quntiles*	-	-	-	-	-
Quintile 1	-	-	-	-	-
Quintile 2	-0.000	-0.000	-0.000	0.000	0.000
Quintile 3	0.000	0.000	0.000	-0.000	-0.000
Quintile 4	0.000	0.000	-0.000	-0.000	-0.000
Quintile 5	-0.000	-0.000	-0.000	0.000	0.000
Total					
Variable	**Coefficient effect – CΔη**				
*Displaced*	**-**	**-**	**-**	**-**	**-**
No	**-**	**-**	**-**	**--**	
Yes	0.004	0.008	0.004	0.028	0.044
*Age group*	**-**	**-**	**-**	**-**	**-**
Less than 18 years	-0.000	-0.001	-0.001	-0.001	-0.002
18 – 44 years	**-**	**-**	**-**	**-**	**-**
45 – 64 years	-0.005	-0.004	-0.008	-0.007	-0.010
65 years or more	-0.002	-0.000	-0.001	-0.002	-0.003
*Gender*	**-**	**-**	**-**	**-**	**-**
Male	**-**	**-**	**-**	**-**	**-**
Female	-0.002	-0.002	0.000	0.003	0.006
*Residence*	**-**	**-**	**-**	**-**	**-**
Urban	**-**	**-**	**-**	**-**	**-**
Rural	-0.010	-0.016	0.009	0.000	-0.028
*Work*	**-**	**-**	**-**	**-**	**-**
Formal	**-**	**-**	**-**	**-**	**-**
Informal	-0.048	-0.027	-0.029	0.013	0.025
Out of Labor Force	0.005	0.007	0.014	0.015	0.015
*Conflict level*	**-**	**-**	**-**	**-**	**-**
Outside of Meta	-0.004	-0.004	-0.003	-0.002	-0.002
Capital city	**-**	**-**	**-**	**-**	**-**
Highly affected	-0.031	-0.034	-0.040	-0.055	-0.071
No conflict	0.004	-0.000	0.001	0.005	0.006
Lowly affected	0.005	-0.002	-0.000	-0.011	-0.013
*Ethnicity*	**-**	**-**	**-**	**-**	**-**
Majority	**-**	**-**	**-**	**-**	**-**
Minority	-0.004	-0.003	-0.003	-0.003	-0.006
*Education*	**-**	**-**	**-**	**-**	**-**
None	**-**	**-**	**-**	**-**	**-**
Primary School	0.017	0.018	0.056	0.027	0.060
Secondary School	-0.009	-0.010	-0.018	-0.019	-0.023
Undergraduate	0.002	-0.002	-0.016	-0.000	-0.007
*Marital status*	**-**	**-**	**-**	**-**	**-**
Married	**-**	**-**	**-**	**-**	**-**
Consensual Union	-0.001	0.000	-0.001	0.006	0.006
Divorced	-0.002	-0.002	-0.001	0.005	0.009
Widow/er	0.002	0.001	0.002	0.003	0.005
Single	-0.000	0.002	0.002	-0.003	-0.001
*Health insurance scheme*	**-**	**-**	**-**	**-**	**-**
Not affiliated	-0.000	0.000	-0.000	0.000	-0.001
Contributive	**-**	**-**	**-**	**-**	**-**
Subsidized	-0.008	-0.027	-0.031	-0.033	-0.068
Other	0.002	0.002	-0.001	0.003	0.002
*Sick in the previous 12 months*	**-**	**-**	**-**	**-**	**-**
Yes	**-**	**-**	**-**	**-**	**-**
No	0.007	0.006	0.009	0.003	0.004
*Children under 5 years old*	0.000	0.000	-0.001	-0.001	-0.001
*Log household size*	0.001	0.001	0.000	0.000	-0.003
*WHODAS*	-0.002	-0.003	-0.002	0.000	0.001
*SRQ+*	**-**	**-**	**-**	**-**	**-**
No	**-**	**-**	**-**	**-**	**-**
Yes	-0.003	-0.003	-0.003	-0.001	-0.008
*Quintiles*	-	-	-	-	-
Quintile 1	-	-	-	-	-
Quintile 2	-0.018	-0.018	-0.009	0.004	0.007
Quintile 3	0.000	0.000	0.000	0.000	0.000
Quintile 4	0.021	0.024	-0.014	-0.042	-0.029
Quintile 5	0.037	0.035	0.008	-0.028	-0.007
Total					

*Source*: Own elaboration based on CONPAS 2014 – 2018

*Abbreviations*: *SRQ+* person has a possible mental health disorder (i.e., a SRQ positive case)

## Discussion

### Main conclusions

Inequalities in catastrophic expenditures increased from a concentration index of -0.152 in 2014 to -0.232 in 2018, meaning that, in both years but more sharply in 2018, it is the population groups at lower socioeconomic levels that are experiencing catastrophic health expenditures more frequently. Our results show that changes in catastrophic expenditure inequalities over the period 2014-2018 are mostly explained by changes in the importance of specific sociodemographic determinants over the same period and, to a much lesser extent, by changes in inequalities in socioeconomic factors. For both years, people living in rural areas, at middle or older age ranges, with health disabilities, being previously sick, or living in conflict-affected municipalities were more prone to experience catastrophic health expenditures than other groups.

### Comparison with Colombian and international studies

Few international studies have explored changes in catastrophic expenditure inequalities in (post-)conflict settings. Edoka et al. [17] analyze changes in catastrophic health expenditures after the end of the eleven-year civil armed conflict in Sierra Leone for the years 2003 to 2011. In contrast to our study, where catastrophic expenditure incidence increased from 29.3 to 30.7% during the pre/post-conflict period examined, Edoka et al. [17] found a decrease from 50 to 32% in the incidence of catastrophic expenditures, which the authors attribute to higher use of regional health facilities instead of NGO health services, a reduction of ill-health, and relocation of households to areas with better living conditions. Nevertheless, post-conflict levels of catastrophic expenditures are similar in both countries (30.7 and 32%). Differences may be attributed to the more severe impairment (to virtual collapse) of health services and infrastructure during the armed conflict in Sierra Leone in comparison to Colombia. This post-conflict recovery of the most basic health services in Sierra Leone from their nearly inexistence, may have contributed to the significant drop in catastrophic expenditure levels in this country . The longer time frame of the Sierra Leone analysis (almost eight years) may further explain the different conclusions of our study, as a consequence of the more extended recovery period for health services. As indicated by our results, major contributors to health inequalities and catastrophic expenditures in Colombia are much more related to individual characteristics (age, mental and physical disabilities, for example) than health service provision or insurance affiliation. These contextual differences may require health policies to be targeted at specific populations to achieve reductions in the overall levels of catastrophic expenditures in Colombia.

Even though catastrophic expenditures remained almost constant (29.3 and 30.7%), and inequalities increased, our results do not necessarily indicate negative outcomes after the Colombian peace treaty. As in Edoka et al. [17], results show how area-related factors (urban/rural household location and conflict incidence) were key for determining changes in health expenditure patterns. Results show higher chances of catastrophic expenditures in highly affected territories only for 2018. Conflict-deescalation and the peace agreement may have facilitated lower-income groups’ access to health services, especially in territories highly impacted by conflict. This, consequently, may have led to higher levels of out-of-pocket expenditures and, therefore, to higher chances of experiencing catastrophic expenditures for this population. Also, conflict de-escalation in post-conflict scenarios may lead, at least in the short run, to a reduction in violent actions and, consequently, facilitate the recovery (or even establishment) of healthcare services in the region.

Consequently, this may lead to quality improvements and favor the provision of healthcare supplies and operation of health professionals in areas where only humanitarian brigades used to provide some healthcare. Simultaneously, conflict de-escalation may facilitate economic recovery in conflict-affected regions, which may improve household income for some populations , reduce financial vulnerability, and, therefore, explain the drop in catastrophic expenditures in 2018 for some of the highest income groups (Quntile 4). Both mechanisms leave levels of catastrophic expenditures inaltered and increase inequality but, ultimately, reflect improvements in health care utilization. Therefore, in conflict-affected territories, two health policy priorities become especially important: first, guarantee the recovery, provision, and maintenance of adequate and permanent health services over time and, second, design better insurance and financial protection policies that guarantee continuous health care utilization at low costs to the most vulnerable groups.

It is noteworthy that the incidence of catastrophic expenditures in the conflict-affected region that we examine (30.7%) is much higher than the average incidence figures for Colombia reported in other studies. Recent estimates from national household surveys point to an incidence of catastrophic spending between 2.2 and 8.2% for Colombia in 2016, depending on the threshold adopted [34]. Amaya-Lara [24] estimates an average incidence of catastrophic health expenditures of 9.6% for a 20% threshold. The importance of the level of conflict violence for explaining health inequalities, found through our decomposition approach, shows how different areas may be exposed to different circumstances that increase households’ risk of incurring catastrophic health payments. This highlights the perils of relying on average figures to set priorities for national health policy, under the risk of masking important inequities against vulnerable groups that deserve urgent attention. The higher chances of catastrophic expenditures to occur in conflict-affected regions and rural areas may be explained by higher difficulties in providing sustained health services in these areas, which may lead to higher care provision costs and limited service capacity, all of which may increase the costs of medical care to families, reality that calls for remedial policies targeted explicitly to these population groups.

### Main contribution, strengths, and limitations of the study

Even though our results apply primarily to the region of Meta, Colombia, this research is based on a large-scale survey that is representative of rural and urban areas exposed to different levels of conflict intensity. This characteristic allows us to draw more general insights about the relationship between inequalities in catastrophic health expenditures and the distribution of socioeconomic characteristics in conflict-affected settings. Crucially, our data's panel structure allows us to estimate changes right before and after one of the most critical episodes in the history of Colombian armed conflict: the signing of the Peace Accord with the FARC guerilla group in 2016. Our research contributes to understanding changes in catastrophic expenditures in post-conflict scenarios, shedding light also into the influence of conflict violence patterns to explain changes in health expenditure inequalities. Rather than establishing causal links between catastrophic expenditure inequalities and the Colombian Peace Agreement, our research focuses on offering more general insights that can assist with designing relevant health policies in a post-conflict period.

Access to health expenditure and other detailed representative data for more than 1000 households has enabled us to perform more fine-grained analyses of the relationship between conflict and catastrophic expenditures, compared to the previous studies focused on specific population groups or cities. Unfortunately, we do not have information on health expenditures for our study population before 2014, which would have been informative to determine how household expenditures evolved during earlier stages of the Colombian conflict. Our analysis is also not geared towards allowing firm conclusions to be drawn about the causal effect of the peace agreement on catastrophic expenditures in Meta, or about long-run trends in health expenditures, due to our methodological strategy and the relatively short period covered by our data. The Oaxaca decomposition method does not allow us to identify when health inequalities started to increase or the role of other unobserved contributing factors that may explain changes in these inequalities, such as personal preferences or risk attitudes.

We found that our quantitative estimates of the distributional effects are robust to changes in the threshold of catastrophic expenditures but less so for the coefficient effects. This may be due in part to possible measurement errors in health expenditures due to recall bias. Even though the survey expenditure questions referring to 2014 and 2018 were the same, longer recall periods may lead to imprecisions in total expenditure figures reported and, usually, will lead to an underestimation of health expenses and/or overestimation of other everyday expenses, like food expenditures, which may, in turn, lead to some imprecision in the estimation of catastrophic expenditures (Beegle et al. 2010). Therefore, these differences may lead to certain imprecisions in the estimation of the concentration indexes, and similarly, in the differences of these indexes between 2014 and 2018 and of inequalities between 2014 and 2018. Further studies, therefore, should analyze the evolution of health expenses during subsequent years to confirm if these expenses continues to rise, or, on the contrary, catastrophic expenditures diminish once health services strengthen in these regions, leading to reduced costs in health provision.

Nevertheless, our catastrophic spending results do seem consistent with those from previous studies (e.g. Edoka et al. [17]), although it must be noted that such studies have generally faced the same limitations in applying a similar estimation methodology to ours, thus highlighting the fact that recall and measurement bias are likely to be present in any analyses of self-reported health expenditures. Due to the specific characteristics and particularities of armed conflicts in several regions of the world, and the state of development of health care systems in territories impacted by conflict, some others variables may be relevant for explaining inequalities in catastrophic expenditures. Therefore, additional studies should be conducted in conflict affected regions to extend our knowledge on the factors that may lead to improvements in the distribution of health care costs among conflict affected populations.

## Conclusion

Health care provision and coverage is a difficult task in any part of the world. However, conflict and its negative consequences in health give origin to major complexities for guaranteeing health care services. Adequate and sustained health coverage is difficult in conflict-affected regions as well as ensuring health equity in outcomes and opportunities. In this order, health policies not only must improve health provision but contribute to minimizing social differences that sustain inequalities and, ultimately, reduce overall wellbeing for some of the most vulnerable populations groups of the world. Post-conflict Colombia is a significant opportunity and scenario to reduce inequalities and promote capacity building in one of the countries where socioeconomic inequalities have historically sustained health care differences.

## Data Availability

The datasets used and/or analyzed during the current study are available from the corresponding author on reasonable request.
